# X Chromosomes' Shape-Shifting Foreshadows Random Inactivation

**DOI:** 10.1371/journal.pbio.0040195

**Published:** 2006-05-09

**Authors:** Françoise Chanut

Molecular biology often seems like it leaves nothing to chance: signal A triggers reaction B, which activates enzyme C and silences gene D—leading cells to divide, differentiate, or die. But chance does sometimes meddle with molecular biology, as shown for instance by the apparently random inactivation (silencing) of the X chromosome. Indeed, in mammals, one copy of the X chromosome becomes silent early in the development of female embryos. As a result, female cells, which have two X chromosomes, produce the same amount of X-encoded molecules as male cells, which have only one. But while male cells have no choice as to which X to keep active, female cells do. That they leave that decision to chance is illustrated by the fact that female tissues are mosaics of cells with either the paternal or the maternal X chromosome in the silent state.

Precisely when chance comes into play during X inactivation is unclear. The prevailing theory is that the X chromosomes of female embryos are equivalent until the onset of inactivation, at which point the chromosomes' fates as active (Xa) or inactive (Xi) become sealed through chance selection. But Susanna Mlynarczyk-Evans, Barbara Panning, and colleagues challenge this theory with a new study that shows that X chromosomes oscillate between two states with different potentials for inactivation before they commit to the Xa or Xi fate. Their observations suggest that random X inactivation reflects a much earlier randomization of X chromosome states than previously thought.

Mlynarczyk-Evans, Panning, and colleagues studied mouse embryonic stem (ES) cells, which mimic in culture the early growing stages of embryonic development, before differentiation triggers X inactivation. Using fluorescent probes specific to various X-encoded genes, the researchers observed that in approximately half of female ES cells, a gene would appear as a single spot (S) on one X and a doublet (D) on the other. Moreover, nearby genes on the same chromosome most often displayed the same S or D status. In any culture of actively dividing cells, one might expect a gene to appear as a singlet before DNA replication, and a doublet after replication. But using a variety of tests, the researchers demonstrated that S signals persisted after X chromosome replication. On the other hand, S signals were sensitive to experimental procedure, and split into two when cells underwent a staining protocol known to disrupt chromosome architecture. The researchers concluded that the X chromosomes of proliferating, undifferentiated ES cells exist in two different physical states.

Curious about what these different states might portend, the researchers repeated their experiments in ES cells carrying various mutations that bias X inactivation. For instance, in cells that harbor a wild-type X and an X mutant for the *Xist* gene, the wild-type X always becomes the Xi. In a large proportion of such cells, the researchers saw that the wild-type X had mostly D signals, and the mutant mostly S signals. Similar biases were observed with other mutations, suggesting that S and D signals (and the different physical states they reveal) might be early markers of the Xi and Xa fate.

But if wild-type X chromosomes are already predisposed for a future Xi or Xa fate before inactivation begins, how is randomness achieved? A possible answer to that question is that pre-inactivation states are unstable. To address this possibility, the researchers established cultures from single ES cells that harbored two wild-type chromosomes, one of which was easily distinguished from the other because it had fused to Chromosome 2. Each founder cell presumably carried either the normal or the fused X in the pre-Xi or pre-Xa state. Yet, in the progeny of each founder, both chromosomes were found in either state in equal proportions. Hence, X chromosomes seem able to switch from one state to the other during cell divisions, quickly achieving a random distribution of the pre-Xi and pre-Xa states.

Mlynarczyk-Evans, Panning, and colleagues do not yet understand the biochemical mechanisms that allow X chromosomes to switch states coordinately in dividing ES cells. But their observations suggest that the differentiation signal that triggers X inactivation might not initiate randomness, but simply stabilize the state of already randomized, shape-shifting chromosomes.

## 

**Figure:**
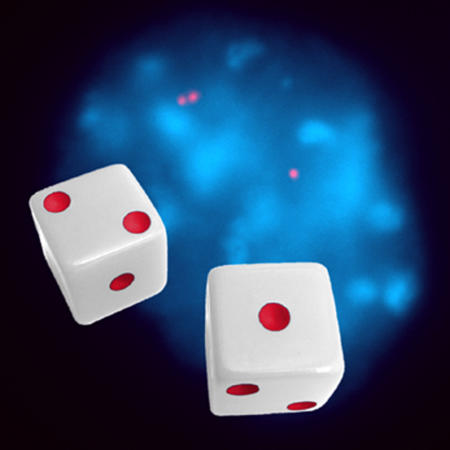
Chromosome-wide differences prior to X inactivation may underlie the random choice of which X chromosome will be silenced.

